# Patients’ recovery of mobility and return to original residence after hip fracture are associated with multiple modifiable components of hospital service organisation: the REDUCE record-linkage cohort study in England and Wales

**DOI:** 10.1186/s12877-023-04038-2

**Published:** 2023-07-27

**Authors:** Rita Patel, Andrew Judge, Antony Johansen, Elsa M. R. Marques, Tim Chesser, Xavier L. Griffin, Muhammad K. Javaid, REDUCE Study Group, Yoav Ben-Shlomo, Celia L. Gregson

**Affiliations:** 1 Musculoskeletal Research Unit, Translational Health Sciences, Bristol Medical School, University of Bristol, Learning and Research Building, Level 1, Southmead Hospital, Bristol, BS10 5NB UK; 2 Nuffield Department of Orthopaedics, Rheumatology and Musculoskeletal Sciences, University of Oxford, Oxford, UK; 3 NIHR Biomedical Research Centre at University Hospitals Bristol and Weston NHS Foundation Trust and the University of Bristol, Bristol, UK; 4 School of Medicine, Cardiff University and University Hospital of Wales, Cardiff, UK; 5 National Hip Fracture Database, Royal College of Physicians, London, UK; 6 Department of Trauma and Orthopaedics, Southmead Hospital, North Bristol NHS Trust, Bristol, UK; 7 Barts Bone and Joint Health, Barts and The London School of Medicine and Dentistry, Queen Mary University of London, London, UK; 8 Royal London Hospital, Barts Health NHS Trust, London, UK; 9 Population Health Sciences, Bristol Medical School, University of Bristol, Bristol, UK; 10 National Institute for Health Research (NIHR) Applied Research Collaboration West (ARC West) at University of Bristol and United Hospitals Bristol NHS Foundation Trust, Bristol, UK; 11 Older People’s Unit, Royal United Hospital NHS Foundation Trust Bath, Combe Park, Bath, UK

**Keywords:** **(3–10)**: Hip - orthopaedic & trauma surgery, Geriatric medicine, Delivery of Health Care, Hospital Services, Fragility fracture

## Abstract

**Background:**

Hip fractures are devastating injuries causing disability, dependence, and institutionalisation, yet hospital care is highly variable. This study aimed to determine hospital organisational factors associated with recovery of mobility and change in patient residence after hip fracture.

**Methods:**

A cohort of patients aged 60 + years in England and Wales, who sustained a hip fracture from 2016 to 2019 was examined. Patient-level Hospital Episodes Statistics, National Hip Fracture Database, and mortality records were linked to 101 factors derived from 18 hospital-level organisational metrics. After adjustment for patient case-mix, multilevel models were used to identify organisational factors associated with patient residence at discharge, and mobility and residence at 120 days after hip fracture.

**Results:**

Across 172 hospitals, 165,350 patients survived to discharge, of whom 163,230 (99%) had post-hospital discharge destination recorded. 18,323 (11%) died within 120 days. Among 147,027 survivors, 58,344 (40%) across 143 hospitals had their residence recorded, and 56,959 (39%) across 140 hospitals had their mobility recorded, at 120 days. Nineteen organisational factors independently predicted residence on hospital discharge e.g., return to original residence was 31% (95% confidence interval, CI:17–43%) more likely if the anaesthetic lead for hip fracture had time allocated in their job plan, and 8–13% more likely if hip fracture service clinical governance meetings were attended by an orthopaedic surgeon, physiotherapist or anaesthetist. Seven organisational factors independently predicted residence at 120 days. Patients returning to their pre-fracture residence was 26% (95%CI:4–42%) more likely if hospitals had a dedicated hip fracture ward, and 20% (95%CI:8–30%) more likely if treatment plans were proactively discussed with patients and families on admission. Seventeen organisational factors predicted mobility at 120 days. More patients re-attained their pre-fracture mobility in hospitals where (i) care involved an orthogeriatrician (15% [95%CI:1-28%] improvement), (ii) general anaesthesia was usually accompanied by a nerve block (7% [95%CI:1-12%], and (iii) bedside haemoglobin testing was routine in theatre recovery (13% [95%CI:6-20%]).

**Conclusions:**

Multiple, potentially modifiable, organisational factors are associated with patient outcomes up to 120 days after a hip fracture, these factors if causal should be targeted by service improvement initiatives to reduce variability, improve hospital hip fracture care, and maximise patient independence.

**Supplementary Information:**

The online version contains supplementary material available at 10.1186/s12877-023-04038-2.

## Background

Hip fracture is the most frequent serious traumatic injury sustained by older people [1]. Following a hip fracture, they often face substantial losses in quality of life [2, 3], functional mobility, and independence, with institutionalisation common [4]. Hip fracture services are provided through complex multidisciplinary organisational structures. National audits such as the National Hip Fracture Database (NHFD) in England, Wales, and Northern Ireland set benchmarking standards in an effort to standardise national performance [5].

Qualitative research has shown that after a hip fracture, patients’ primary concerns centre on whether they will get back to their pre-injury residence, i.e. ‘home’, and whether they will be able to walk as well as they did previously [6]. In England and Wales, mobility recovery and the need for institutionalised care following a hip fracture varies substantially between hospitals [1, 2, 7–9]. In 2020, of 63,284 patients with hip fracture, 70% had returned to their original residence by 120 days, but this varied from 41 to 90% in different hospitals [10]. This variation may partly be explained by patient case-mix, but we hypothesise that organisational structures in the delivery of hip fracture services contributed to this variation. Understanding how variation in care delivery affects patient outcomes can inform service-level interventions to reduce unwarranted variation and maximise health equity and patient independence.

This study used a large record-linkage cohort of nearly all hip fracture patients in England and Wales, combining anonymised individual patients’ data with measures of how well hospitals deliver hip fracture care as measured by publicly available service audits and reports. The study aimed to determine which hospital-level organisational factors are associated with better patient outcomes, specifically the ability to return home and to recover mobility after a hip fracture.

## Methods

### Study cohort

The REDUCE (REducing unwarranted variation in the Delivery of high-qUality hip fraCture services in England and Wales) study analysed all index hip fracture cases in adults aged 60 years or older admitted to an English or Welsh hospital between 1st April 2016 and 31st March 2019 (i.e., the first occurrence of hip fracture for a patient within the 3-year study period). Hip fractures were included regardless of whether or not surgery was performed. Anonymised patient-level data for index hip fracture cases were obtained from the routinely collected Hospital Episodes Statistics (HES) Admitted Patient Care database for National Health Service (NHS) hospitals in England, and for hospitals in Wales from its counterpart the Patient Episode Database for Wales (PEDW). Data were linked by NHS Digital to Office for National Statistics (ONS) Civil Registration Deaths data [11, 12], and the resulting HES-ONS and PEDW-ONS data extracts linked to the National Hip Fracture Database (NHFD) (Additional Fig. 1). The NHFD is a Healthcare Quality Improvement Partnership clinical audit, capturing data on hip fracture care from all NHS hospitals in England, Wales, and Northern Ireland [13]. Hip fracture admissions were identified using the International Classification of Diseases, Tenth Revision (ICD-10) codes for fracture of head and neck of femur (S72.0), trochanteric fracture (S72.1), subtrochanteric fracture (S72.2), and unspecified fracture of femur (S72.9) [14]. Second hip fractures during the study period were excluded, to avoid double-counting, as it was not possible to distinguish with certainty between two separate hip fracture events in HES and for these fractures the prognosis would differ.

**Fig. 1:**
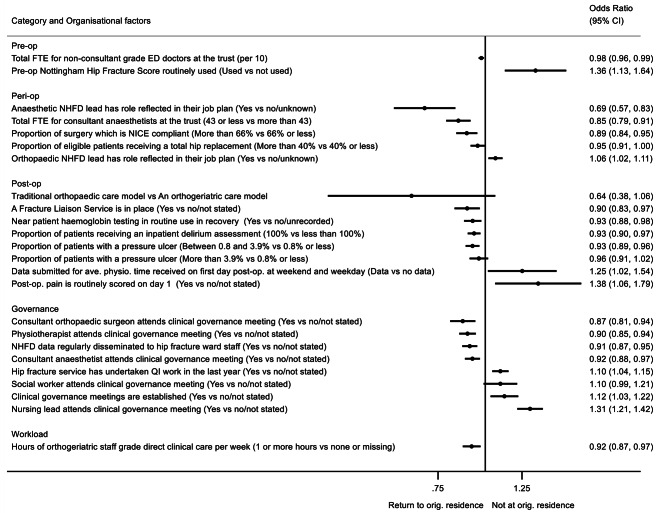
The effect of organisational factors on residence status at discharge, after accounting for patient case-mix OR > 1 indicates more likely to not return to original residence Organisational factors adjusted for case-mix (age, sex, ASA classification, hip fracture type, pre-fracture residence, and pre-fracture mobility) and mutually adjusted for all backward selected factors shown in Additional Table 1. Factors with p-value < 0.1 shown. N = 163,230 ASA = American Society of Anesthesiologists, ave.=average, CI = confidence interval, ED = emergency department, FTE = full time equivalent, NHFD = National Hip Fracture Database, NICE = National Institute for Clinical Excellence, op = operative, orig.=original, physio.=physiotherapy, QI = quality improvement, trust = a National Health Service hospital that is managed by a trust

**Table 1:** Characteristics of N = 165,350 patients discharged following hip fracture in England and Wales (2016–2019) by outcome

		Post-fracture outcomes recorded												
		**Residence at discharge**				**Residence at 120 days**				**Mobility at 120 days**				
**N patients**		**163,230**				**58,344**				**56,959**				
** N hospital**		**172**				**143**				**140**				
		**Return to original residence or better**		**Not returned to original residence**		**Return to original residence or better**		**Not returned to original residence**		**Same/** **improved mobility**		**Worse mobility**		
**Pre-fracture characteristics**		**N**	**%**	**N**	**%**	**N**	**%**	**N**	**%**	**N**	**%**	**N**	**%**	
Total		117,384	72	45,846	28	49,308	85	9,036	15	25,589	45	31,370	55	
Age (years)		Mean = 81.5, SD = 8.8		Mean = 84.8, SD = 7.6		Mean = 81.5, SD = 8.5		Mean = 85.4, SD = 7.6		Mean = 81.5, SD = 8.9		Mean = 82.5, SD = 8.1		
Age (years)	60–69	13,423	86	2,045	13	5,200	94	341	6	2,975	55	2,401	45	
	70–79	30,960	79	7,889	20	13,054	90	1,429	10	6,550	46	7,609	54	
	80–89	50,790	68	22,879	31	22,455	84	4,365	16	11,081	42	15,128	58	
	90+	22,211	62	13,033	36	8,599	75	2,901	25	4,983	44	6,232	56	
Sex	Female	84,705	71	32,548	27	36,136	85	6,491	15	18,918	45	22,719	55	
ASA grade*	I & II	35,339	80	8,685	20	15,907	93	1,259	7	7,400	44	9,372	56	
	III	65,367	68	28,912	30	27,611	82	5,945	18	14,575	44	18,195	56	
	IV & V	16,678	66	8,249	33	5,790	76	1,832	24	3,614	49	3,803	51	
Hip fracture type	Intracapsular	72,560	74	24,075	25	29,970	86	4,916	14	16,128	47	17,977	53	
	Inter, subtrochanteric or other	44,824	66	21,771	32	19,338	82	4,120	18	9,461	41	13,393	59	
Pre-fracture residence	Own home/sheltered housing	92,564	68	41,962	31	41,754	85	7,638	15	20,669	43	27,538	57	
	Not from own home	24,820	86	3,884	13	7,554	84	1,398	16	4,920	56	3,832	44	
Pre-fracture mobility	Freely mobile without walking aids	50,801	79	12,559	20	21,708	91	2,069	9	6,421	28	16,866	72	
	Mobile outdoors with 1 or 2 aids or frame	39,858	65	20,363	33	16,727	83	3,447	17	7,428	38	12,247	62	
	Some indoor, or no functional, mobility	26,725	66	12,924	32	10,873	76	3,520	24	11,740	84	2,257	16	

ASA = American Society of Anesthesiologists. SD = standard deviation

*I & II (healthy patient or patient with mild systemic disease), III (patient with a severe but not incapacitating systemic disease), IV & V (a patient with an incapacitating disease that is also life threatening or a moribund patient not expected to live for 24 h with or without surgery)

Rounding means that some percentages may not total 100

### Patient outcomes

Patient outcomes were:

(i) residence at discharge from hospital,
(ii) residence 120 days after presentation with hip fracture and
(iii) mobility 120 days after presentation with hip fracture.

The NHFD records mobility and residence outcomes at 120 days, as patient recovery plateaus at this timepoint [3]. Residence was derived from NHFD records of residence prior to admission (recorded as own home/sheltered housing, residential care, or nursing care), and at discharge and 120 days (recorded as own home/sheltered housing, residential care, nursing care, rehabilitation unit, acute hospital, other, or dead). Those reported as dead during initial hospital admission were excluded, and for 120 day outcomes those who died prior to 120 days follow-up were excluded. All available data were used (even if 120-day outcomes were collected more than 120 days after admission). The classification of residence (at discharge or 120 days) ranked own home/sheltered housing as ‘best’, then residential care, and nursing care as ‘worst’. If residence post fracture was the same or ‘better’ compared to residence at admission, this was coded as 0=‘yes, returned to original residence (or better)’. The remaining destinations, e.g., still in an acute hospital or inpatient rehabilitation unit, were coded 1=‘no, not returned to original residence (moved to rehabilitation)’. This classification is different from that used by the NHFD, which reports return to the same residence within 120 days (yes or no) [10]. The NHFD records pre-fracture and post-fracture mobility at 120 days (‘freely mobile without aids’, ‘mobile outdoors with one aid’, ‘mobile outdoors with two aids or frame’, ‘some indoor mobility but never goes outside without help’, ‘no functional mobility [using lower limbs]’). Mobility data were classified as 0=‘same/improved mobility’ and 1=‘decline in mobility (worse mobility)’. Less independent residence or mobility status was coded as ‘1’, so that odds ratios less than one indicate a desirable or better outcome, and odds ratios greater than one indicate an undesirable or worse outcome. This coding was selected to be analogous to mortality outcome reporting.

### Patient-level case-mix data

The same case-mix adjustment was used as that for the NHFD clinical audit [15]. This comprised age (in 10-year bands from 60 to 90 years and over), sex, American Society of Anesthesiologists’ (ASA) classification of pre-operative physical status [16] (I & II; III; IV & V), hip fracture type (intracapsular; trochanteric, subtrochanteric or other), and as mentioned above: pre-fracture residence (own home/sheltered housing; other) and pre-fracture mobility (freely mobile without aids; mobile outdoors with 1 or 2 aids or frame; some indoor mobility or no functional mobility) [15]. Hence, we controlled for pre-fracture residence and mobility.

### Organisational-level factors

Service data at the hospital level included national audits, data series, and ratings, much of which are publicly available [12]. Using data from 18 multidisciplinary audits and reports, and combining English and Welsh data where possible, 231 organisational factors were identified, which characterised care delivery within the hip fracture pathway from admission to discharge. Expert consensus review determined that at least 101 organisational factors were potentially relevant to the outcomes of residence and mobility [12].

Organisational factors potentially related to one or more patient outcomes were mapped to 14 domains of hip fracture care by expert consensus, using a systematic approach as previously described [12]. The domains were emergency department (ED), anaesthetic, orthopaedic and orthogeriatric services, admission volume, nutrition assessment, delirium prevention, pain management, ward staffing/care, therapy provision, rehabilitation, inpatient falls, hospital governance, and hip fracture service governance [12]. Clinical governance is defined as the system through which NHS organisations are accountable for continuously improving the quality of their services and safeguarding high standards of care. Each organisational factor was further assigned to a single over-arching theme (pre-, peri-, or post-operative care, governance, or workload). Where organisational-level factors were time specific, they were linked to patient-level data using hospital codes, and the year (and month/quarter if available) corresponding to the date of hip fracture admission.

### Patient and public involvement and engagement (PPIE)

The REDUCE Study PPIE group comprised four individuals with osteoporosis and/or a history of hip fracture. This group contributed to part of the REDUCE ethics application, study design, and materials and analysis approach, and their responses to a prioritisation exercise of key organisational factors and patient outcomes informed this analysis.

### Statistical analysis

Multilevel regression models were used to estimate the associations between organisation-level factors and the three patient-level outcomes. The hierarchical data structure comprised patients (level 1), nested within hospitals (level 2). Using C-statistics, the proportion of variance in each patient outcome that was due to patient-level characteristics (i.e., case-mix), and the between-hospital variability attributable to fixed organisational effects (i.e., the proportion of between-hospital variance explained by service configuration) were quantified. Organisational factors were dichotomised, categorised if more than two groups or, for continuous measures, converted to linear splines at quartiles (or tertiles as appropriate). Backward stepwise elimination identified the organisational factors most strongly associated with each outcome. Factors were simplified by expert review and splines were dichotomised/categorised at threshold(s) or converted back to continuous measures on an appropriate scale. Missing outcome data were not imputed, instead the potential for artefactual statistical associations potentially attributable to missing data were investigated and associations presented or described. Statistical analyses were performed in Stata 16.1 (StataCorp LP, College Station, TX, USA) and MLwiN v3.04 (Centre for Multilevel Modelling, University of Bristol, UK). The STROBE (Strengthening the Reporting of Observational Studies in Epidemiology) guideline was used to report this study [17].

## Results

After excluding patients who died during their initial hospital admission, we included 165,350 patients who presented with a hip fracture to one of the 172 hospitals between 2016 and 2019. Hospital discharge destination was recorded for 163,230 patients (99%) after a median length of stay of 15 days (interquartile range, 9–26 days). At 120 days after injury, 18,323 (11%) patients had died. Of the 147,027 who were still alive, 58,344 (40%) across 143 hospitals had their place of residence recorded, and 56,959 (39%) across 140 hospitals had their mobility recorded. Overall, 56,832 patients had data available for all three residence and mobility outcomes (see Additional Fig. 2). Older patients, those with poorer pre-fracture mobility, with higher ASA grades, and with extra-capsular fractures, tended to be discharged to rehabilitation, and were less likely to have returned to their original residence at 120 days (Table 1). Older patients and those with extra-capsular fractures had poorer mobility at 120 days. The model fit for each patient outcome was improved by adjusting for patient case-mix but was unchanged by the addition of organisational factors or domains of hip fracture care (e.g., C-statistic for residence at discharge was 0.66 [95% confidence interval, CI: 0.66, 0.67], which increased to 0.76 [95%CI: 0.76, 0.76] with the addition of case-mix, and remained unchanged with the further addition of organisational factors). Below, we report the organisational factors with the strongest evidence (where effect estimates do not cross the null) supporting associations with residence and mobility outcomes adjusted for case-mix, and mutually adjusted for all selected organisational factors. A summary of organisational factors associated with positive patient outcomes is provided in Table 2.

**Fig. 2:**
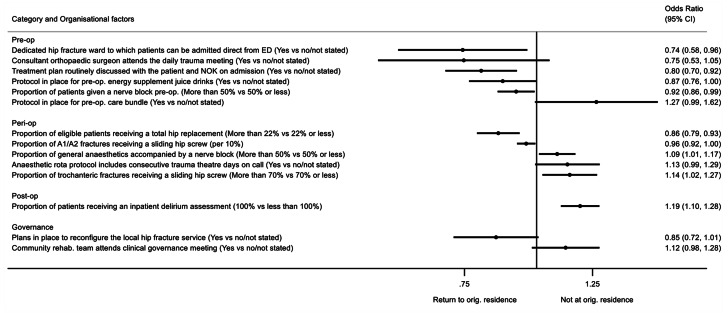
The effect of organisational factors on residence status at 120 days, after accounting for patient case-mix OR > 1 indicates more likely to not return to original residence Organisational factors adjusted for case-mix (age, sex, ASA classification, hip fracture type, pre-fracture residence, and pre-fracture mobility) and mutually adjusted for all backward selected factors shown in Additional Table 2. Factors with p-value < 0.1 shown. N = 58,344 A1 = stable, A2 = unstable, ASA = American Society of Anesthesiologists, CI = confidence interval, ED = emergency department, NOK = next of kin, op = operative, orig.=original, rehab.=rehabilitation, trust = a National Health Service hospital that is managed by a trust

**Table 2:** Summary of selected findings

Greater likelihood of discharge to original residence in hospitals with:	
Pre-op	More FTE for non-consultant grade ED doctors at the trust
Peri-op	Anaesthetic NHFD lead with role reflected in their job plan
	More FTE for consultant anaesthetists at the trust
	Greater proportion of surgery which is NICE compliant
Post-op	A Fracture Liaison Service is in place
	Near-patient haemoglobin testing in routine use in recovery*
	100% of patients receive an inpatient delirium assessment
Governance	Consultant orthopaedic surgeon attends clinical governance meeting
	Physiotherapist attends clinical governance meeting
	NHFD data regularly disseminated to hip fracture ward staff*
	Consultant anaesthetist attends clinical governance meeting
Workload	More hours of orthogeriatric staff grade direct clinical care per week is provided
**Greater likelihood of return to original residence at 120 days in hospitals with**:	
Pre-op	Dedicated hip fracture ward to which patients can be admitted direct from ED
	Treatment plan routinely discussed with the patient and NOK on admission
	Larger proportion of patients given a nerve block pre-op.
Peri-op	Larger proportion of eligible patients receive a total hip replacement
**Greater likelihood of good patient mobility outcomes in hospitals with**:	
Pre-op	Pre-op. pain is routinely scored
	More FTE for ED consultants at the trust
Peri-op	100% of patients assessed by an orthogeriatrician within 72 h of admission
	Anaesthetic rota protocol includes consecutive trauma theatre days on call
	Larger proportion general anaesthetics accompanied by a nerve block
Post-op	Near-patient haemoglobin testing in routine use in recovery*
Governance	Nursing lead attends clinical governance meeting
	NHFD data regularly disseminated to hip fracture ward staff*
	Clinical governance meetings occur monthly
Workload	More hours of orthogeriatric consultant direct clinical care per week is provided

* indicates this organisational factor is important for more than one outcome

ED = Emergency Department, FTE = full time equivalent, NHFD = National Hip Fracture Database, NICE = National Institute for Clinical Excellence, NOK = next of kin, op = operative

### Residence at discharge from hospital

Twenty-three organisational factors were independently associated with patient residence immediately post discharge, of which 19 factors had 95% confidence intervals independent of the null (Fig. 1 and Additional Table 1). Higher proportions of patients were discharged home (rather than to rehabilitation) from hospitals where the NHFD anaesthetic lead had this role reflected in their job plan, where hospitals had fewer than 43 full-time equivalent (FTE) anaesthetists, and where a higher proportion of National Institute for Health and Care Excellence (NICE)-compliant hip fracture surgery was performed [8].

Postoperatively, the presence of a Fracture Liaison Service integrated into the hospital services, routine bedside haemoglobin testing in theatre recovery, and routine delirium assessment were all independently associated with a higher chance of home discharge, albeit with small effect sizes. Orthopaedic, physiotherapy and anaesthetic staff attendance at clinical governance meetings was associated with an increase in the chance of patients returning home of 13% [95%CI: 6, 19%], 10% [95%CI: 6, 15%] and 8% [95%CI: 3, 12%], respectively, whereas attendance by nursing leads was associated with a 31% [95%CI:21, 42%] greater chance of discharge to rehabilitation. Hospitals that regularly disseminated NHFD clinical audit data to staff were more likely to discharge patients directly home, as were those delivering more hours of staff grade-level orthogeriatric care.

In contrast, the routine use of outcome prediction scores (i.e., the Nottingham Hip Fracture Score [18, 19]) and pain scores, submission of data regarding availability of weekend and weekday physiotherapy (these data provided *versus* no data provided), and the recent need to conduct quality improvement (QI) work (QI undertaken in last year *versus* no QI/not stated) were each associated with a greater likelihood of transfer to a rehabilitation facility.

### Residence at 120 days post-injury

Fourteen organisational factors independently predicted residence status at 120 days, of which seven factors had 95% confidence intervals that did not cross the null (Fig. 2 and Additional Table 2). Hospitals with a dedicated hip fracture ward to which patients can be transferred directly from the ED had a 26% increased likelihood of patients getting home at 120 days (95%CI: 4%, 42%); 64.6% of patients (n = 106,742) attended hospitals which reported having such a ward. Routine discussion on admission about treatment plans with the patient and those important to them was strongly associated with patients returning home by 120 days (20% [95%CI: 8%, 30%]). Greater use of (usually femoral or fascia-iliaca) nerve blocks preoperatively (e.g. in the ED) was associated with 8% (95%CI: 1%, 14%) increased likelihood of patients getting home.

A strong independent surgical predictor of being home at 120 days was having been treated in a hospital where greater than 22% of eligible hip fracture patients received a total hip replacement (14% [95%CI: 7%, 21%]). In contrast, hospitals where general anaesthesia was more commonly accompanied by a nerve block, where a higher proportion of sliding hip screws were used for trochanteric fractures, and where delirium assessment was said to be performed in 100% of instances, were all associated with a reduced likelihood of being at home at 120 days.

### Recovery of mobility at 120 days post injury

**Fig. 3:**
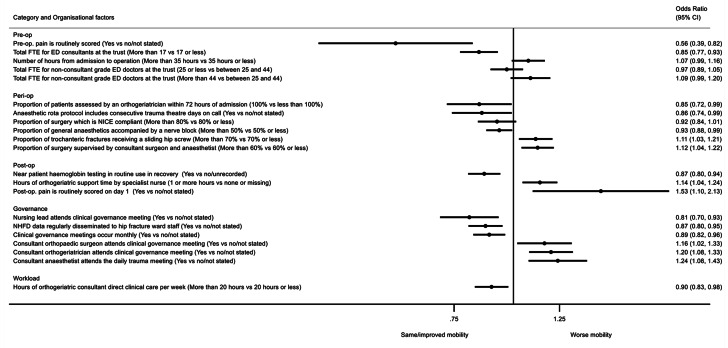
The effect of organisational factors on post-fracture mobility at 120 days, after accounting for patient case-mix OR > 1 indicates more likely to have worse mobility compared with pre-injury mobility levels (collected at the time of hospital admission) Organisational factors adjusted for case-mix (age, sex, ASA classification, hip fracture type, pre-fracture residence, and pre-fracture mobility) and mutually adjusted for all backward selected factors shown in Additional Table 3. Factors with p-value < 0.1 shown. N = 56,959 ASA = American Society of Anesthesiologists, CI = confidence interval, ED = emergency department, FTE = full time equivalent, NHFD = National Hip Fracture Database, NICE = National Institute for Clinical Excellence, op = operative, trust = a National Health Service hospital that is managed by a trust

Twenty organisational factors independently predicted mobility at 120 days, of which 17 factors had 95% confidence intervals independent of the null (Fig. 3 and Additional Table 3). Having a greater number of emergency medicine consultants in the hospital was associated with a 15% (95%CI: 7%, 23%) greater likelihood of recovery of patient mobility. Similarly, there was a greater (15% [95%CI: 1%, 28%]) chance of patients recovering mobility if they were managed in a hospital where all patients were assessed by an orthogeriatrician within 72 h of admission or where anaesthetic rotas included consecutive theatre days for trauma on call (14% [95%CI: 1%, 26%]). Greater provision of nerve blocks at the time of general anaesthesia was associated with improved mobility (7% [95%CI: 1%, 12%]).

Postoperatively, routine use of bedside haemoglobin testing in theatre recovery was associated with a 13% (95%CI: 6%, 20%) greater likelihood of recovery of patient mobility. Where clinical governance meetings were attended by nursing leads, there was a 19% (95%CI: 7%, 30%) greater chance of mobility recovery. Disseminating NHFD audit data to hip fracture staff and holding clinical governance meetings monthly were both independently associated with good mobility outcomes. There was an additional independent association between a higher number of hours of direct clinical care provided by consultant orthogeriatricians (rather than specialist nurses) and mobility recovery.

In contrast, where surgery more often needed senior supervision by a consultant surgeon and consultant anaesthetist, mobility outcomes were poorer. Orthopaedic surgeon and orthogeriatrician attendance at clinical governance meetings were associated with poorer mobility outcomes. Also, having the consultant anaesthetist attend the daily trauma meeting was associated with risk of worse mobility (24% [95%CI: 8%, 43%]). Furthermore, inconsistent results were found for the use of pain scores; hospitals which routinely scored patients’ pre-operative pain had a 44% (95%CI: 18%, 61%) greater chance of patients recovering mobility at 120 days, whilst hospitals routinely scoring pain on day one postoperatively saw a 53% (95%CI: 10%, 113%) increased risk of worse mobility compared with hospitals where pain scores were either not performed or not reported; however, these appeared as a result of case-mix adjustment (Additional Table 3).

### Investigation of missing data at 120 days

Patient-level pre-injury residence and mobility (with other case-mix variables) were examined to see if they predicted missingness in 120-day outcome data (Additional Table 4). Little evidence was seen to suggest that missingness was associated with 120-day outcomes other than a suggestion that those ‘mobile outdoors with 1 or 2 aids or frame’ pre-operatively were more likely to have missing data. For each organisational factor, the percentage of missing data for each outcome relative to available outcome data was examined. This showed that for some organisational factors the proportion of missing data was high; and potentially differential, with the organisational factor having up to 13% more missing data than the comparison category in the worst case (Additional Tables 5 and 6). However, for patient-level mortality status at 120 days, no organisational factor was differentially missing for either residence or mobility at 120 days; differences in missingness of data were negligible (Additional Tables 5 and 6). The proportion of patients returning to their original residence at 120 days (amongst those with available data) against the proportion missing 120-day residence data in each hospital are presented in Additional Fig. 3; no clear association was seen (0.02, 95% CI: -0.02, 0.06). A similar plot for mobility presents the proportion of patients with good mobility outcomes per hospital against the proportion missing mobility data; no clear association was seen (0.05, 95% CI: -0.03, 0.12) (Additional Fig. 4).

## Discussion

After accounting for case-mix and the multi-level structure of the data, this study has identified a range of organisational factors, many potentially amenable to change, associated with important patient outcomes. Direct admission to a specialist hip fracture ward, recognition of the importance of discussing treatment plans on admission with patients and the people important to them, pre-operative use of nerve blocks, and provision of total hip replacements to those who are eligible, were all independently associated with successfully returning patients to their original residence by 120 days. Mobility recovery by 120 days was higher in hospitals with more emergency department consultants, and in hospitals where care involved an orthogeriatrician, where pain was routinely scored pre-operatively, where general anaesthesia was usually accompanied by a nerve block, and where clinical governance meetings were held each month. Clinical governance was an important domain influencing all three outcomes, although effect sizes and associations were not always consistent. Clinical governance meeting attendance by orthopaedic, anaesthetic and physiotherapy leads was associated with an increased likelihood of discharge from hospital to original residence, whilst nursing attendance was associated with improved mobility recovery at 120 days. Routine point-of-care haemoglobin testing in theatre recovery was associated with both direct discharge home and mobility recovery at 120 days, as was the routine practice of feeding back local hip fracture audit data to staff teams caring for hip fracture patients. Patients were more likely to be discharged to their original residence if the NHFD anaesthetic lead role was reflected in anaesthetic job plans. As approximately 60% of patients were not followed up at 120 days, findings for 120-day outcomes should be interpreted with caution. However, this study does provide insights into organisational factors potentially important for patient recovery that, if causal, could be targeted by service improvement initiatives to reduce variability in healthcare delivery. We encourage clinical services to improve 120-day data collection as local and national audits provide valuable data, otherwise not captured by other routine data systems, which provide an efficient method to appraise healthcare in a real-world setting.

Few studies have examined multiple organisational factors across England and Wales over a significant timespan to identify factors potentially influencing patient outcomes. The findings that patients were more likely to be discharged home from hospitals where anaesthetic lead roles had time assigned may reflect that clarity in job planning improves ownership of clinical responsibility, increases team communication, and assigns time for governance activities [20, 21]. Clinical governance aims to improve the quality and safety of services [22]; the attendance of orthopaedic and anaesthetic consultants and physiotherapists at these meetings is key to integrating a multidisciplinary hip fracture care pathway [23, 24]. Our findings suggest nursing attendance at clinical governance meetings may translate to fewer discharges directly home, but rather to rehabilitation facilities, to improve mobility recovery by 120 days. This may reflect management of bed pressures and/or different rehabilitation perspectives from other members of the multidisciplinary team, or alternatively only on well-staffed wards are nurses able to attend clinical governance meetings. Multiple barriers to monthly multidisciplinary meetings have been documented and should be tackled to encourage attendance and full participation from all members of the hip fracture team [25, 26]. Fewer patients were discharged directly home from hospitals that routinely used pre-operative outcome prediction and post-operative pain scores. Whilst this may appear unexpected, it could be explained if pre-operative scores are used to determine post-operative rehabilitation intensity and bespoke patient-centred discharge planning is lost, and if pain management protocols prompt more analgesia than is required, such that sedative side effects hamper recovery of inpatient mobility. Furthermore, the ability to report data on the availability of inpatient physiotherapy services and the recent conduct of hip fracture service QI work were associated with fewer direct discharges home. These factors may indicate hospitals that have recognised a service inefficiency and have taken action to collect data/ address the issue.

Several relatively simple, readily modifiable organisational factors have been identified that can be introduced/established in all UK hospitals which may improve patient care. Routine point-of-care haemoglobin testing in theatre recovery requires a small initial equipment investment and is easily incorporated into routine recovery protocols with minimal training. Anticipation of postoperative anaemia can enable prompt transfusion, where appropriate, which can increase the success of first day mobilisation in the context of frailty [27]. Local audit data are readily available from the NHFD and routine feedback to hip fracture team staff is highly feasible. These data, routinely reviewed at clinical governance meetings, can easily be communicated to teams, with a little planning. Individuals who feel they are informed members of a multidisciplinary team are motivated to provide higher quality patient care [28]. We have previously shown greater provision of pre-operative nerve blocks (usually femoral or fascia-iliaca) – which reduces opiate analgesic use and associated side effects [29] – is associated with shorter lengths of hospital stay [30]; here we add an increased likelihood of patients being back home at 120 days to the associated benefits. These findings support the NHFD key performance indicator (KPI-0) encouraging the prompt provision of local anaesthetic nerve blocks during admission [31].

Discussion of the treatment plan, and therefore risks and expected outcomes, with the patient and their family/friends at admission was associated with patients being more likely to return home within 120 days. Evidence shows that patients and carers are frequently unclear of treatment plans, highlighting the value of clear two-way communication [32]. In this analysis, 58 hospitals – managing during the study period a total of 52,004 hip fracture patients – were not routinely discussing treatment plans when audited; a substantial oversight in care [33]. Previous studies have found that hospitals with a dedicated hip fracture ward have increased success returning patients home at discharge and one year [34], with strong evidence that dedicated hip fracture wards lead to improved 30-day survival [35].

More patients returned home by 120 days from hospitals where a high proportion of total hip replacements were performed for those who were eligible. This may reflect subtle residual differences in case-mix, since total hip replacement is recommended by NICE guidelines for those with good mobility, no cognitive impairment, and anaesthetic fitness [8]; or alternatively a difference in the availability and attitudes of senior orthopaedic surgeons [36]. Consistent with this finding, more patients were discharged home from hospitals where a high proportion of NICE-compliant surgery (following the national guidelines for surgical treatment) was performed. In hospitals where more surgery was supervised by a consultant surgeon and anaesthetist, an association with worse patient mobility was seen; potentially reflecting residual confounding by more complex patient case-mix necessitating senior supervision, or else hospitals where operations were performed by more junior surgeons/anaesthetists, necessitating more senior surgeon/anaesthetic supervision, but with poorer mobility recovery. During admission, adequate staffing of emergency departments, particularly in terms of consultants, could mean shorter waits before transfer of patients to specialist wards, improving the quality of care. Alternatively, it could be that hospitals with larger emergency departments and therefore more consultants have better patient mobility outcomes. Similarly, the ability to routinely record patients’ pre-operative pain scores may be indicative of improved emergency department organisation and efficiency (rather than pain management alone), all of which may combine to improve mobility outcomes. We and others have shown the benefits of post-hip fracture inpatient physiotherapy on short-term patient recovery [30, 37], supporting prompt mobilisation as a key performance indicator of hip fracture care [31], yet despite this, we know that most patients who survive a hip fracture do not recover their pre-fracture independence [38]. Aside from physiotherapy attendance at clinical governance meetings, other inpatient physiotherapy metrics were not identified in our analyses of mobility recovery at 120 days, which may be explained by the fact that post-discharge community physiotherapy has a greater role in determining mobility recovery by 120-days. We lacked data on post-discharge rehabilitation and care provision; however, at the time of writing it is known to be highly variable in its delivery across the UK. We were only able to examine organisational factors which had been metricised across all hip fracture hospitals, with few data available from post discharge NHS rehabilitation facilities. Currently in the UK, whilst hospital-level healthcare data are a rich resource, community-based and social care data collection is not well established, thus data linkage between health and social care data is not possible, as it is in many Scandinavian countries. Thus, our understanding of post-discharge community-based rehabilitation is poor. Since the study dates, this has been worsened by the increasing role of non-NHS intermediate care providers.

This study has a series of strengths, having systematically linked hip fractures treated in the NHS across England and Wales to follow-up data for multiple outcomes over the 120-day period post hip fracture. The subsequent linkage of such a dataset to comprehensive organisational national datasets was labour intensive and is unique. The longitudinal study period of 3 years offered an opportunity to allow for temporal variations within hospitals and to give a more representative overall estimate for each hospital. A study strength is the multilevel data analysis, i.e. patients nested in hospitals are accounted for, enabling a true measure of association at the hospital level and the generation of standard errors which account for clustering. The study also integrated clinical expert consensus to determine *a-priori* appropriate organisational factors to include in the models and verify the face-validity of findings.

Despite the value of such a large national dataset, this study has limitations. Selection bias may influence the reporting of 120-day outcomes, as more than two thirds of patients were not followed up, potentially due to hospitals being unable (due to workload pressures) or unwilling to conduct follow-up data collection, or patients being uncontactable if they have moved or if unable/unwilling to respond. Fitter patients may be more able to respond to telephone, or sometimes letter, follow up. There was little evidence of survival bias when examining organisational factors against 120-day mortality.

The level of missing outcome data, up to 13% for some organisational factors, means these analyses are vulnerable to selection (“collider”) bias which could attenuate or reverse true causal associations, potentially explaining counter-intuitive associations if both the exposure and outcomes predict whether someone is included in the analysis, for example explaining the inconsistent findings relating to clinical governance participants for discharge vs. 120-day outcomes. We analysed the first occurrence of hip fracture within the 3-year study period, thus whilst some patients may have had a prior contralateral hip fracture and been included, those with a subsequent contralateral hip fracture during follow-up would have been included as well (although the outcomes following any second contralateral hip fracture in the study period did not contribute to analysis); this may have involved approximately 5% of hip fracture patients, with associated poorer recovery outcomes [39].

Large sample sizes can generate associations that appear important statistically, but which may not be clinically meaningful. Causality cannot be inferred from these observational data, which are prone to type 1 error. It was too computationally intensive to use bootstrapping to repeat the stepwise variable selection to provide internal validation of multilevel models. It was not possible to independently validate the – albeit generally high-quality – organisational datasets, although some were troubled by missing data and hence certain factors either could not be used or missing data were included as a category. The multi-level model fit was improved by adjusting for patient case-mix but not by the addition of organisational factors, which may reflect the ecological nature of the organisational data; that multiple aspects of hospital care could not be measured (e.g. multiple ward moves); or simply the lack of sensitivity the C-statistic has, as a rank-based measure, compared to likelihood-based modelling [40]. The study design may be prone to the ecological fallacy, as organisational factors are measured at the hospital level, so associations may not apply to the patient level, e.g., mean number of hours from admission to operation; although in some cases factors truly reflect a hospital-level rather than a patient-level exposure, e.g. governance measures. Furthermore, organisational factors may not be independent at the hospital level, as they may cluster by the overall quality of a hospital.

## Conclusions

Hip fractures lead to pain, disability and loss of independence. This study used a wide range of audit and report data to characterise hospital services providing hip fracture care and to examine associations between organisational characteristics and key aspects of recovery: patients’ residence and mobility after hip fracture. Although limited by missing outcome data, this study provides insights into multiple, potentially modifiable, organisational factors associated with patient residence and mobility status. Many reflect the multidisciplinary approach to hip fracture care. These findings if causal provide a range of potential targets for interventions that could reduce variability and improve the quality of hip fracture services and ultimately patient outcomes.

## Electronic supplementary material

Below is the link to the electronic supplementary material.

Supplementary Material 1

## Data Availability

Electronic health records are, by definition, considered sensitive data in the UK by the Data Protection Act and cannot be shared via public deposition because of information governance restriction in place to protect patient confidentiality. Access to data is available once approval has been obtained through the individual constituent entities controlling access to the data. Researchers interested in accessing: (i) HES data can apply for access through NHS Digital’s Data Access Request Service (DARS) https://dataaccessrequest.hscic.gov.uk/; (ii) PEDW data can apply for access through the NHS Informatics Service – PEDW Data Online https://nwis.nhs.wales/information-services/health-intelligence/pedw-data-online/; and (iii) NHFD data can apply for access through the Falls and Fragility Fracture Audit Programme (FFFAP) Expression of interest form https://www.rcplondon.ac.uk/file/fffap-expression-interest-form-1/ and a Data Access Request Form (DARF) https://www.rcplondon.ac.uk/guidelines-policy/applying-work-falls-and-fragility-fracture-audit-programme-data/.

## Declarations

### Data declaration

This publication is based on data collected by or on behalf of the Healthcare Quality Improvement Partnership, who have no responsibility or liability for the accuracy, currency, reliability and/or correctness of this publication.

## Abbreviations

ASA: American Society of Anesthesiologists
CI: confidence interval
ED: Emergency Department
FFFAP: Falls and Fragility Fracture Audit Programme
FTE: Full-time equivalent
HES: Hospital Episodes Statistics
HQIP: Healthcare Quality Improvement Partnership
ICD: International Classification of Diseases
KPI: Key Performance Indicators
NHFD: National Hip Fracture Database
NHS: National Health Service
NICE: National Institute for Health and Care Excellence
NWIS: NHS Wales Informatics Service
ONS: Office for National Statistics
PEDW: Patient Episode Database for Wales
PPIE: Patient and public involvement and engagement
QI: Quality Improvement
REDUCE: REducing unwarranted variation in the Delivery of high-qUality hip fraCture services in England and Wales
STROBE: Strengthening the Reporting of Observational Studies in Epidemiology
