# Supplementary material for: Patients’ recovery of mobility and return to original residence after hip fracture are associated with multiple modifiable components of hospital service organisation: the REDUCE record-linkage cohort study in England and Wales
Source: BMC Geriatr. 2023 Jul 27;23:459. doi: 10.1186/s12877-023-04038-2 (PMC10375618; doi:10.1186/s12877-023-04038-2)
Supplement: Supplementary file 1 — Supplementary Material 1 [file 12877_2023_4038_MOESM1_ESM.docx]

# Additional file

Additional table 1: The effect of organisational factors on residence status at discharge, after accounting for patient case-mix (N=163,230)

|  | Organisational factor (range or categories in brackets) | Organisational factor adjusted for case mix and other factors, OR (95%CI)* | | p-value | Data source | Organisational factor adjusted for case-mix, OR (95% CI) | Organisational factor without case-mix adjustment, OR (95% CI) |
| --- | --- | --- | --- | --- | --- | --- | --- |
|  | **Factors with p-value<0.1** |  |  |  |  |  |  |
| Pre-op | Total FTE for non-consultant grade ED doctors at the trust (per 10) | 0.98 | (0.96, 0.99) | 0.003 | Workforce | 0.99 (0.97, 1) p=0.07 | 0.99 (1, 0.98) p=0.46 |
|  | Pre-op Nottingham Hip Fracture Score routinely used (Used vs not used) | 1.36 | (1.13, 1.64) | 0.001 | NHFD FA | 1.35 (1.12, 1.63) p=0.002 | 1.3 (1.63, 1.09) p=0.003 |
| Peri-op | Anaesthetic NHFD lead has role reflected in their job plan (Yes vs no/unknown) | 0.69 | (0.57, 0.83) | <0.001 | NHFD FA | 0.72 (0.6, 0.87) p=0.001 | 0.74 (0.87, 0.63) p=0.001 |
|  | Total FTE for consultant anaesthetists at the trust (43 or less vs more than 43) | 0.85 | (0.79, 0.91) | <0.001 | Workforce | 0.88 (0.82, 0.94) p<0.001 | 0.89 (0.94, 0.83) p<0.001 |
|  | Proportion of surgery which is NICE compliant (More than 66% vs 66% or less) | 0.89 | (0.84, 0.95) | 0.001 | KPI | 0.9 (0.85, 0.96) p=0.001 | 0.93 (0.96, 0.88) p=0.01 |
|  | Proportion of eligible patients receiving a total hip replacement (More than 40% vs 40% or less) | 0.95 | (0.91, 1.00) | 0.033 | NHFD Charts | 0.97 (0.93, 1.01) p=0.16 | 0.99 (1.01, 0.95) p=0.61 |
|  | Orthopaedic NHFD lead has role reflected in their job plan (Yes vs no/unknown) | 1.06 | (1.02, 1.11) | 0.004 | NHFD FA | 1.02 (0.98, 1.06) p=0.38 | 1.02 (1.06, 0.98) p=0.25 |
| Post-op | Traditional orthopaedic care model vs An orthogeriatric care model | 0.64 | (0.38, 1.06) | 0.084 | NHFD FA | 0.71 (0.43, 1.19) p=0.19 | 0.73 (1.19, 0.46) p=0.2 |
|  | A Fracture Liaison Service is in place (Yes vs no/not stated) | 0.9 | (0.83, 0.97) | 0.005 | FLS-DB | 0.89 (0.82, 0.96) p=0.002 | 0.91 (0.96, 0.85) p=0.01 |
|  | Near-patient haemoglobin testing in routine use in recovery (Yes vs no/unrecorded) | 0.93 | (0.88, 0.98) | 0.004 | NHFD FA | 0.94 (0.9, 0.99) p=0.03 | 0.96 (0.99, 0.92) p=0.13 |
|  | Proportion of patients receiving an inpatient delirium assessment (100% vs less than 100%) | 0.93 | (0.90, 0.97) | 0.001 | BP | 0.98 (0.95, 1.01) p=0.25 | 0.96 (1.01, 0.93) p=0.03 |
|  | Proportion of patients with a pressure ulcer (Between 0.8 and 3.9% vs 0.8% or less) | 0.93 | (0.89, 0.96) | <0.001 | NHFD Charts | 0.94 (0.91, 0.97) p<0.001 | 0.94 (0.97, 0.91) p<0.001 |
|  | Proportion of patients with a pressure ulcer (More than 3.9% vs 0.8% or less) | 0.96 | (0.91, 1.02) | 0.174 | NHFD Charts | 1.05 (1, 1.09) p=0.03 | 1.03 (1.09, 1) p=0.09 |
|  | Data submitted for ave. physio. time received on first day post-op. at weekend and weekday (Data vs no data) | 1.25 | (1.02, 1.54) | 0.035 | PHFSA | 1.19 (0.96, 1.47) p=0.11 | 1.2 (1.47, 0.99) p=0.06 |
|  | Post-op. pain is routinely scored on day 1 (Yes vs no/not stated) | 1.38 | (1.06, 1.79) | 0.016 | NHFD FA | 1.39 (1.08, 1.8) p=0.01 | 1.3 (1.8, 1.03) p=0.03 |
| Governance | Consultant orthopaedic surgeon attends clinical governance meeting (Yes vs no/not stated) | 0.87 | (0.81, 0.94) | <0.001 | NHFD FA | 1.01 (0.97, 1.05) p=0.57 | 1 (1.05, 0.97) p=0.86 |
|  | Physiotherapist attends clinical governance meeting (Yes vs no/not stated) | 0.9 | (0.85, 0.94) | <0.001 | NHFD FA | 0.96 (0.92, 0.99) p=0.01 | 0.97 (0.99, 0.93) p=0.05 |
|  | NHFD data regularly disseminated to hip fracture ward staff (Yes vs no/not stated) | 0.91 | (0.87, 0.95) | <0.001 | NHFD FA | 0.93 (0.89, 0.96) p<0.001 | 0.94 (0.96, 0.9) p=0.001 |
|  | Consultant anaesthetist attends clinical governance meeting (Yes vs no/not stated) | 0.92 | (0.88, 0.97) | 0.001 | NHFD FA | 0.95 (0.91, 0.98) p=0.002 | 0.96 (0.98, 0.93) p=0.02 |
|  | Hip fracture service has undertaken QI work in the last year (Yes vs no/not stated) | 1.1 | (1.04, 1.15) | <0.001 | NHFD FA | 1.05 (1, 1.1) p=0.04 | 1.05 (1.1, 1) p=0.04 |
|  | Social worker attends clinical governance meeting (Yes vs no/not stated) | 1.1 | (0.99, 1.21) | 0.078 | NHFD FA | 1.05 (0.95, 1.15) p=0.32 | 1.03 (1.15, 0.94) p=0.47 |
|  | Clinical governance meetings are established (Yes vs no/not stated) | 1.12 | (1.03, 1.22) | 0.007 | NHFD FA | 1.07 (1.01, 1.14) p=0.03 | 1.06 (1.14, 1) p=0.04 |
|  | Nursing lead attends clinical governance meeting (Yes vs no/not stated) | 1.31 | (1.21, 1.42) | <0.001 | NHFD FA | 1.08 (1.04, 1.13) p<0.001 | 1.07 (1.13, 1.03) p=0.001 |
| Workload | Hours of orthogeriatric staff grade direct clinical care per week (1 or more hours vs none or missing) | 0.92 | (0.87, 0.97) | 0.003 | NHFD FA | 0.89 (0.84, 0.94) p<0.001 | 0.9 (0.94, 0.86) p<0.001 |
|  | **Factors with p-value>=0.1** |  |  |  |  |  |  |
| Pre-op | Treatment plan routinely discussed with the patient and NOK on admission (Yes vs no/not stated) | 0.96 | (0.78, 1.17) | 0.654 | NHFD FA | 1.01 (0.83, 1.24) p=0.89 | 1.03 (1.24, 0.86) p=0.73 |
|  | Total FTE for ED consultants at the trust (More than 12 vs 12 or less) | 0.97 | (0.91, 1.02) | 0.241 | Workforce | 0.97 (0.92, 1.02) p=0.26 | 0.99 (1.02, 0.94) p=0.61 |
|  | Total no. emergency admissions from ED within the hospital trust each month (More than 3000 vs 3000 or less) | 1 | (0.95, 1.06) | 0.912 | ED | 1 (0.95, 1.05) p=0.92 | 1.01 (1.05, 0.96) p=0.57 |
| Peri-op | Proportion of A1/A2 fractures receiving a sliding hip screw (More than 75% vs 75% or less) | 0.99 | (0.94, 1.05) | 0.79 | NHFD Charts | 0.99 (0.94, 1.04) p=0.68 | 0.99 (1.04, 0.95) p=0.72 |
|  | Total FTE for non-consultant grade anaesthetic doctors at the trust (More than 40 vs 40 or less) | 0.99 | (0.94, 1.05) | 0.799 | Workforce | 0.98 (0.92, 1.03) p=0.41 | 0.98 (1.03, 0.93) p=0.52 |
|  | Proportion of trochanteric fractures receiving a sliding hip screw (More than 70% vs 70% or less) | 1 | (0.95, 1.06) | 0.881 | NHFD Charts | 0.99 (0.95, 1.04) p=0.67 | 0.99 (1.04, 0.95) p=0.72 |
|  | Proportion of surgery supervised by consultant surgeon and anaesthetist (Between 40 and 80% vs 40% or less) | 0.97 | (0.92, 1.02) | 0.255 | Benchmark | 0.95 (0.91, 0.99) p=0.006 | 0.96 (0.99, 0.93) p=0.03 |
|  | Proportion of surgery supervised by consultant surgeon and anaesthetist (More than 80% vs 40% or less) | 1.06 | (0.98, 1.14) | 0.132 | Benchmark | 1.09 (1.04, 1.16) p=0.001 | 1.09 (1.16, 1.03) p=0.002 |
| Post-op | Proportion of patients not delirious post-op (More than 75 vs 75 or less) | 0.97 | (0.92, 1.02) | 0.219 | KPI | 0.96 (0.91, 1) p=0.05 | 0.99 (1, 0.94) p=0.51 |
| Governance | Consultant anaesthetist attends the daily trauma meeting (Yes vs no/not stated) | 0.86 | (0.72, 1.04) | 0.118 | NHFD FA | 0.9 (0.74, 1.09) p=0.27 | 0.9 (1.09, 0.75) p=0.23 |
| Workload | Mean number of T&O beds occupied per day (More than 50 vs 50 or less) | 0.99 | (0.93, 1.05) | 0.641 | Beds | 0.99 (0.94, 1.05) p=0.78 | 0.99 (1.05, 0.93) p=0.62 |

OR>1 indicates more likely to not return to original residence.

*Organisational factors are adjusted for case-mix (age, sex, ASA classification, hip fracture type, pre-fracture residence, and pre-fracture mobility) and mutually adjusted for all backward selected factors in table.

A1=stable, A2=unstable, ASA=American Society of Anesthesiologists, Ave.=average, CI=confidence interval, ED=Emergency Department, FA=Facilities Audit, FTE=full time equivalent, NHFD=National Hip Fracture Database, NICE=National Institute for Clinical Excellence, NOK=next of kin, op=operative, physio.=physiotherapy, QI=quality improvement, T&O=Trauma and orthopaedic.

Additional table 2: The effect of organisational factors on residence status at 120 days, after accounting for patient case-mix (N=58,344)

|  | Organisational factor (range or categories in brackets) | Organisational factor adjusted for case mix and other factors, OR (95%CI)* | | p-value | Data source | Organisational factor adjusted for case-mix, OR (95% CI) | Organisational factor without case-mix adjustment, OR (95% CI) |
| --- | --- | --- | --- | --- | --- | --- | --- |
|  | **Factors with p-value<0.1** |  |  |  |  |  |  |
| Pre-op | Dedicated hip fracture ward to which patients can be admitted direct from ED (Yes vs no/not stated) | 0.74 | (0.58, 0.96) | 0.025 | NHFD FA | 0.9 (0.79, 1.03) p=0.12 | 0.89 (1.03, 0.79) p=0.06 |
|  | Consultant orthopaedic surgeon attends the daily trauma meeting (Yes vs no/not stated) | 0.75 | (0.53, 1.05) | 0.096 | NHFD FA | 1.11 (0.84, 1.47) p=0.48 | 1.19 (1.47, 0.91) p=0.19 |
|  | Treatment plan routinely discussed with the patient and NOK on admission (Yes vs no/not stated) | 0.8 | (0.70, 0.92) | 0.002 | NHFD FA | 0.86 (0.76, 0.98) p=0.02 | 0.87 (0.98, 0.77) p=0.02 |
|  | Protocol in place for pre-op. energy supplement juice drinks (Yes vs no/not stated) | 0.87 | (0.76, 1.00) | 0.049 | NHFD FA | 0.93 (0.82, 1.05) p=0.23 | 0.96 (1.05, 0.85) p=0.51 |
|  | Proportion of patients given a nerve block pre-op. (More than 50% vs 50% or less) | 0.92 | (0.86, 0.99) | 0.03 | NHFD Charts | 0.9 (0.84, 0.97) p=0.005 | 0.93 (0.97, 0.87) p=0.03 |
|  | Protocol in place for pre-op. care bundle (Yes vs no/not stated) | 1.27 | (0.99, 1.62) | 0.057 | NHFD FA | 1.1 (0.89, 1.37) p=0.37 | 1.11 (1.37, 0.91) p=0.32 |
| Peri-op | Proportion of eligible patients receiving a total hip replacement (More than 22% vs 22% or less) | 0.86 | (0.79, 0.93) | <0.001 | NHFD Charts | 0.86 (0.79, 0.93) p<0.001 | 0.87 (0.93, 0.8) p=0.001 |
|  | Proportion of A1/A2 fractures receiving a sliding hip screw (per 10%) | 0.96 | (0.92, 1.00) | 0.027 | NHFD Charts | 1 (0.97, 1.03) p=0.82 | 1 (1.03, 0.97) p=0.86 |
|  | Proportion of general anaesthetics accompanied by a nerve block (More than 50% vs 50% or less) | 1.09 | (1.01, 1.17) | 0.027 | NHFD Charts | 1.04 (0.97, 1.12) p=0.28 | 1.06 (1.12, 0.99) p=0.12 |
|  | Anaesthetic rota protocol includes consecutive trauma theatre days on call (Yes vs no/not stated) | 1.13 | (0.99, 1.29) | 0.062 | NHFD FA | 1.04 (0.92, 1.18) p=0.53 | 1.04 (1.18, 0.92) p=0.56 |
|  | Proportion of trochanteric fractures receiving a sliding hip screw (More than 70% vs 70% or less) | 1.14 | (1.02, 1.27) | 0.017 | NHFD Charts | 1.12 (1.03, 1.22) p=0.01 | 1.1 (1.22, 1.01) p=0.03 |
| Post-op | Proportion of patients receiving an inpatient delirium assessment (100% vs less than 100%) | 1.19 | (1.10, 1.28) | <0.001 | BP | 1.22 (1.14, 1.3) p<0.001 | 1.21 (1.3, 1.14) p<0.001 |
| Governance | Plans in place to reconfigure the local hip fracture service (Yes vs no/not stated) | 0.85 | (0.72, 1.01) | 0.061 | NHFD FA | 0.89 (0.76, 1.06) p=0.19 | 0.89 (1.06, 0.76) p=0.15 |
|  | Community rehab. team attends clinical governance meeting (Yes vs no/not stated) | 1.12 | (0.98, 1.28) | 0.092 | NHFD FA | 1.14 (1, 1.3) p=0.04 | 1.14 (1.3, 1.01) p=0.04 |
|  | **Factors with p-value>=0.1** |  |  |  |  |  |  |
| Pre-op | T&O manager attends the daily trauma meeting (Yes vs no/not stated) | 0.87 | (0.70, 1.08) | 0.213 | NHFD FA | 1.01 (0.81, 1.26) p=0.93 | 1.05 (1.26, 0.85) p=0.67 |
|  | Protocol in place for pre-op. fluid management (Protocol reported vs no protocol reported) | 0.89 | (0.71, 1.10) | 0.288 | NHFD FA | 0.95 (0.78, 1.15) p=0.61 | 0.97 (1.15, 0.81) p=0.75 |
|  | Total FTE for non-consultant grade ED doctors at the trust (More than 45 vs 45 or less) | 0.97 | (0.86, 1.09) | 0.621 | Workforce | 0.9 (0.82, 1) p=0.06 | 0.89 (1, 0.81) p=0.02 |
|  | Proportion of patients receiving a nutrition assessment during admission (100% vs less than 100%) | 0.98 | (0.91, 1.04) | 0.482 | BP | 1.07 (1.01, 1.14) p=0.02 | 1.08 (1.14, 1.01) p=0.01 |
|  | Number of hours from admission to operation (More than 35 hours vs 35 hours or less) | 0.98 | (0.89, 1.10) | 0.778 | NHFD Charts | 0.91 (0.83, 0.99) p=0.03 | 0.91 (0.99, 0.84) p=0.03 |
|  | Total no. emergency admissions from ED within the hospital trust each month (Between 3000 and 5000 vs 3000 or less) | 0.99 | (0.89, 1.09) | 0.804 | ED | 1.02 (0.95, 1.09) p=0.58 | 1.02 (1.09, 0.95) p=0.66 |
|  | Total no. emergency admissions from ED within the hospital trust each month (More than 5000 vs 3000 or less) | 0.96 | (0.83, 1.10) | 0.554 | ED | 0.91 (0.83, 1) p=0.05 | 0.92 (1, 0.84) p=0.08 |
|  | Proportion of patients admitted to orthopaedic ward within 4 hours of presentation to the ED (More than 50 vs 50 or less) | 1.02 | (0.94, 1.10) | 0.633 | Benchmark | 1.05 (0.98, 1.13) p=0.17 | 1.03 (1.13, 0.96) p=0.39 |
|  | Protocol in place for pre-op. nerve blocks (Yes vs no/not stated) | 1.11 | (0.84, 1.45) | 0.465 | NHFD FA | 1.09 (0.85, 1.41) p=0.5 | 1.11 (1.41, 0.87) p=0.39 |
| Peri-op | Total FTE for non-consultant grade T&O doctors at the trust (More than 26 vs 26 or less) | 0.96 | (0.87, 1.05) | 0.372 | Workforce | 0.95 (0.87, 1.03) p=0.23 | 0.93 (1.03, 0.86) p=0.1 |
|  | Total FTE for consultant anaesthetists at the trust (per 10) | 0.99 | (0.96, 1.01) | 0.364 | Workforce | 0.99 (0.97, 1.01) p=0.23 | 0.99 (1.01, 0.97) p=0.16 |
|  | Total FTE for non-consultant grade anaesthetic doctors at the trust (More than 40 vs 40 or less) | 1.01 | (0.91, 1.13) | 0.815 | Workforce | 0.96 (0.87, 1.05) p=0.33 | 0.94 (1.05, 0.86) p=0.21 |
|  | Total FTE for T&O consultant surgeons at the trust (More than 13 vs 13 or less) | 1.02 | (0.90, 1.17) | 0.713 | Workforce | 0.98 (0.88, 1.09) p=0.66 | 0.97 (1.09, 0.88) p=0.62 |
|  | Proportion of patients receiving surgery within 36 hours of admission (More than 70% vs 70% or less) | 1.08 | (0.98, 1.19) | 0.132 | BP | 1.14 (1.05, 1.23) p=0.002 | 1.14 (1.23, 1.05) p=0.001 |
| Post-op | Patients in hospital receive physiotherapy on Saturday and/or Sunday (Yes vs no weekend physio) | 0.93 | (0.80, 1.07) | 0.286 | NHFD FA | 1.03 (0.9, 1.18) p=0.64 | 1.04 (1.18, 0.91) p=0.54 |
|  | Near-patient haemoglobin testing in routine use in recovery (Yes vs no/unrecorded) | 0.96 | (0.87, 1.05) | 0.35 | NHFD FA | 0.96 (0.89, 1.05) p=0.37 | 0.97 (1.05, 0.9) p=0.5 |
|  | Part of hip fracture ward staffed as a high dependency area (Yes vs no/not stated) | 1 | (0.84, 1.19) | 0.999 | NHFD FA | 1.01 (0.84, 1.21) p=0.94 | 1.07 (1.21, 0.89) p=0.48 |
|  | No. of days between discharge and start of community therapy reported (Yes vs not reported) | 1.01 | (0.87, 1.19) | 0.859 | PHFSA | 1.07 (0.94, 1.23) p=0.31 | 1.09 (1.23, 0.96) p=0.17 |
|  | Return to ward care bundle in place (Yes vs no/not stated) | 1.03 | (0.89, 1.18) | 0.729 | NHFD FA | 1.02 (0.89, 1.18) p=0.77 | 1.02 (1.18, 0.9) p=0.74 |
|  | Proportion of patients promptly mobilised () | 1.04 | (0.93, 1.16) | 0.506 | KPI | 1.05 (0.94, 1.16) p=0.41 | 1.04 (1.16, 0.94) p=0.44 |
|  | Proportion of patients promptly mobilised (More than 80% vs Between 70 and 80%) | 1.03 | (0.94, 1.13) | 0.463 | KPI | 0.97 (0.89, 1.05) p=0.46 | 0.96 (1.05, 0.88) p=0.3 |
|  | Data submitted for average physiotherapy time (minutes) received in first week post-op. (Data vs no data) | 1.05 | (0.77, 1.43) | 0.752 | PHFSA | 1.01 (0.87, 1.17) p=0.91 | 1.05 (1.17, 0.91) p=0.5 |
|  | Hours of orthogeriatric support time by specialist nurse (1 or more hours vs none or missing) | 1.05 | (0.96, 1.15) | 0.272 | NHFD FA | 1.02 (0.94, 1.12) p=0.63 | 1.04 (1.12, 0.96) p=0.36 |
|  | Hospital submitted PHFSA audit data (Any vs none/missing) | 1.08 | (0.78, 1.47) | 0.652 | PHFSA | 1.01 (0.86, 1.18) p=0.93 | 1.03 (1.18, 0.88) p=0.72 |
|  | Patients in hospital receive routine orthogeriatric review on Saturday and/or Sunday (Yes vs no/not stated) | 1.11 | (0.95, 1.31) | 0.196 | NHFD FA | 1.05 (0.89, 1.25) p=0.56 | 1.07 (1.25, 0.91) p=0.39 |
|  | No. WTE physios (trained or assistants) on duty today (Data submitted vs no data submitted) | 1.15 | (0.91, 1.46) | 0.244 | NHFD FA | 0.92 (0.81, 1.04) p=0.18 | 0.89 (1.04, 0.79) p=0.06 |
|  | Model of care (Another model of care vs traditional model of orthopaedic care) | 0.79 | (0.45, 1.38) | 0.408 | NHFD FA | 0.73 (0.58, 0.92) p=0.009 | 0.67 (0.92, 0.54) p<0.001 |
|  | Model of care (Post-operative geriatric care vs traditional model of orthopaedic care) | 1.4 | (0.80, 2.44) | 0.237 | NHFD FA | 1.03 (0.79, 1.34) p=0.83 | 1 (1.34, 0.78) p=0.99 |
|  | Model of care (Routine orthogeriatric review vs traditional model of orthopaedic care) | 1.28 | (0.78, 2.09) | 0.328 | NHFD FA | 1.18 (1.04, 1.34) p=0.009 | 1.18 (1.34, 1.05) p=0.006 |
|  | Model of care (Shared care/Admitted under geriatrician vs traditional model of orthopaedic care) | 1.1 | (0.67, 1.81) | 0.702 | NHFD FA | 0.92 (0.81, 1.04) p=0.18 | 0.95 (1.04, 0.84) p=0.35 |
| Governance | Clinical governance meetings are established (Yes vs no/not stated) | 0.93 | (0.78, 1.11) | 0.423 | NHFD FA | 0.98 (0.84, 1.14) p=0.82 | 1.02 (1.14, 0.88) p=0.79 |
|  | T&O manager attends clinical governance meeting (Yes vs no/not stated) | 0.95 | (0.88, 1.03) | 0.201 | NHFD FA | 0.97 (0.9, 1.04) p=0.37 | 0.98 (1.04, 0.92) p=0.62 |
|  | Number of NAIF data items missing (Any missing vs none missing) | 0.96 | (0.84, 1.10) | 0.563 | NAIF | 1.03 (0.91, 1.16) p=0.69 | 1 (1.16, 0.89) p=0.93 |
|  | Consultant anaesthetist attends clinical governance meeting (Yes vs no/not stated) | 1.03 | (0.95, 1.12) | 0.486 | NHFD FA | 0.97 (0.9, 1.05) p=0.42 | 0.99 (1.05, 0.92) p=0.81 |
| Workload | Hours of orthogeriatric consultant direct clinical care per week (Between 12 and 20 hours vs 12 hours or less) | 0.91 | (0.82, 1.02) | 0.107 | NHFD FA | 1 (0.92, 1.08) p=0.92 | 1.01 (1.08, 0.93) p=0.81 |
|  | Hours of orthogeriatric consultant direct clinical care per week (More than 20 hours vs 12 hours or less) | 0.93 | (0.83, 1.05) | 0.251 | NHFD FA | 0.97 (0.89, 1.05) p=0.44 | 0.96 (1.05, 0.88) p=0.3 |
|  | Proportion of hip fractures occurring in inpatients (More than 4% vs 4% or less) | 1.05 | (0.99, 1.12) | 0.109 | NHFD Charts | 1.08 (1.01, 1.15) p=0.02 | 1.08 (1.15, 1.02) p=0.008 |

OR>1 indicates more likely to not return to original residence.

*Organisational factors are adjusted for case-mix (age, sex, ASA classification, hip fracture type, pre-fracture residence, and pre-fracture mobility) and mutually adjusted for all backward selected factors in table.

A1=stable, A2=unstable, ASA=American Society of Anesthesiologists, CI=confidence interval, ED=Emergency Department, FA=Facilities Audit, FTE=full time equivalent, NHFD=National Hip Fracture Database, NOK=next of kin, op=operative, rehab.=rehabilitation, T&O=Trauma and orthopaedic, WTE=whole time equivalent.

Additional table 3: The effect of organisational factors on post-fracture mobility at 120 days, after accounting for patient case-mix (N=56,959)

|  | Organisational factor (range or categories in brackets) | Organisational factor adjusted for case mix and other factors, OR (95%CI)* | | p-value | Data source | Organisational factor adjusted for case-mix, OR (95% CI) | Organisational factor without case-mix adjustment, OR (95% CI) |
| --- | --- | --- | --- | --- | --- | --- | --- |
|  | **Factors with p-value<0.1** |  |  |  |  |  |  |
| Pre-op | Pre-op. pain is routinely scored (Yes vs no/not stated) | 0.56 | (0.39, 0.82) | 0.003 | NHFD FA | 0.81 (0.65, 1.02) p=0.08 | 0.97 (1.02, 0.79) p=0.77 |
|  | Total FTE for ED consultants at the trust (More than 17 vs 17 or less) | 0.85 | (0.77, 0.93) | <0.001 | Workforce | 0.9 (0.83, 0.98) p=0.01 | 0.93 (0.98, 0.87) p=0.04 |
|  | Number of hours from admission to operation (More than 35 hours vs 35 hours or less) | 1.07 | (0.99, 1.16) | 0.078 | NHFD Charts | 1.03 (0.95, 1.11) p=0.5 | 1.03 (1.11, 0.96) p=0.4 |
|  | Total FTE for non-consultant grade ED doctors at the trust (25 or less vs between 25 and 44) | 0.97 | (0.89, 1.05) | 0.421 | Workforce | 0.95 (0.88, 1.03) p=0.22 | 0.94 (1.03, 0.88) p=0.07 |
|  | Total FTE for non-consultant grade ED doctors at the trust (More than 44 vs between 25 and 44) | 1.09 | (0.99, 1.20) | 0.091 | Workforce | 1.01 (0.93, 1.11) p=0.79 | 1.02 (1.11, 0.94) p=0.7 |
| Peri-op | Proportion of patients assessed by an orthogeriatrician within 72 hours of admission (100% vs less than 100%) | 0.85 | (0.72, 0.99) | 0.038 | BP | 0.8 (0.69, 0.93) p=0.004 | 0.88 (0.93, 0.77) p=0.06 |
|  | Anaesthetic rota protocol includes consecutive trauma theatre days on call (Yes vs no/not stated) | 0.86 | (0.74, 0.99) | 0.038 | NHFD FA | 0.93 (0.81, 1.08) p=0.35 | 0.94 (1.08, 0.83) p=0.39 |
|  | Proportion of surgery which is NICE compliant (More than 80% vs 80% or less) | 0.92 | (0.84, 1.01) | 0.093 | KPI | 0.98 (0.89, 1.07) p=0.63 | 0.97 (1.07, 0.9) p=0.41 |
|  | Proportion of general anaesthetics accompanied by a nerve block (More than 50% vs 50% or less) | 0.93 | (0.88, 0.99) | 0.032 | NHFD Charts | 0.9 (0.85, 0.96) p=0.001 | 0.92 (0.96, 0.88) p=0.003 |
|  | Proportion of trochanteric fractures receiving a sliding hip screw (More than 70% vs 70% or less) | 1.11 | (1.03, 1.21) | 0.009 | NHFD Charts | 1.2 (1.11, 1.29) p<0.001 | 1.11 (1.29, 1.04) p=0.001 |
|  | Proportion of surgery supervised by consultant surgeon and anaesthetist (More than 60% vs 60% or less) | 1.12 | (1.04, 1.22) | 0.003 | Benchmark | 1.1 (1.02, 1.18) p=0.01 | 1.04 (1.18, 0.97) p=0.28 |
| Post-op | Near-patient haemoglobin testing in routine use in recovery (Yes vs no/unrecorded) | 0.87 | (0.80, 0.94) | <0.001 | NHFD FA | 0.85 (0.79, 0.91) p<0.001 | 0.89 (0.91, 0.84) p=0.001 |
|  | Hours of orthogeriatric support time by specialist nurse (1 or more hours vs none or missing) | 1.14 | (1.04, 1.24) | 0.003 | NHFD FA | 1.1 (1.02, 1.19) p=0.02 | 1.04 (1.19, 0.97) p=0.3 |
|  | Post-op. pain is routinely scored on day 1 (Yes vs no/not stated) | 1.53 | (1.10, 2.13) | 0.012 | NHFD FA | 0.98 (0.8, 1.2) p=0.86 | 1.02 (1.2, 0.85) p=0.85 |
| Governance | Nursing lead attends clinical governance meeting (Yes vs no/not stated) | 0.81 | (0.70, 0.93) | 0.003 | NHFD FA | 0.95 (0.88, 1.03) p=0.22 | 0.95 (1.03, 0.89) p=0.12 |
|  | NHFD data regularly disseminated to hip fracture ward staff (Yes vs no/not stated) | 0.87 | (0.80, 0.95) | 0.001 | NHFD FA | 0.87 (0.81, 0.94) p<0.001 | 0.93 (0.94, 0.87) p=0.02 |
|  | Clinical governance meetings occur monthly (Yes vs no/not stated) | 0.89 | (0.82, 0.96) | 0.003 | NHFD FA | 0.86 (0.8, 0.93) p<0.001 | 0.93 (0.93, 0.88) p=0.03 |
|  | Consultant orthopaedic surgeon attends clinical governance meeting (Yes vs no/not stated) | 1.16 | (1.02, 1.33) | 0.026 | NHFD FA | 1.02 (0.94, 1.1) p=0.62 | 1 (1.1, 0.93) p=0.96 |
|  | Consultant orthogeriatrician attends clinical governance meeting (Yes vs no/not stated) | 1.2 | (1.08, 1.33) | 0.001 | NHFD FA | 1.05 (0.97, 1.13) p=0.21 | 1.01 (1.13, 0.94) p=0.88 |
|  | Consultant anaesthetist attends the daily trauma meeting (Yes vs no/not stated) | 1.24 | (1.08, 1.43) | 0.003 | NHFD FA | 1.23 (1.06, 1.41) p=0.005 | 1.06 (1.41, 0.93) p=0.38 |
| Workload | Hours of orthogeriatric consultant direct clinical care per week (More than 20 hours vs 20 hours or less) | 0.9 | (0.83, 0.98) | 0.01 | NHFD FA | 0.92 (0.85, 0.99) p=0.03 | 0.95 (0.99, 0.89) p=0.17 |
|  | **Factors with p-value>=0.1** |  |  |  |  |  |  |
| Pre-op | Proportion of patients admitted to orthopaedic ward within 4 hours of presentation to the ED (More than 50 vs 50 or less) | 0.95 | (0.88, 1.02) | 0.144 | Benchmark | 0.97 (0.91, 1.04) p=0.39 | 0.99 (1.04, 0.94) p=0.79 |
|  | Total no. emergency admissions from ED within the hospital trust each month (More than 4000 vs 4000 or less) | 1.02 | (0.93, 1.12) | 0.623 | ED | 1 (0.91, 1.09) p=0.93 | 1.03 (1.09, 0.95) p=0.49 |
| Peri-op | Proportion of A1/A2 fractures receiving a sliding hip screw (More than 90% vs 90% or less) | 1.02 | (0.95, 1.09) | 0.541 | NHFD Charts | 1.04 (0.97, 1.1) p=0.3 | 0.98 (1.1, 0.93) p=0.44 |
|  | Orthopaedic NHFD lead has role reflected in their job plan (Yes vs no/unknown) | 1.02 | (0.95, 1.10) | 0.531 | NHFD FA | 0.99 (0.93, 1.06) p=0.86 | 1.01 (1.06, 0.95) p=0.81 |
| Post-op | Proportion of patients not delirious post-op (More than 75 vs 75 or less) | 1.03 | (0.95, 1.11) | 0.479 | KPI | 1.02 (0.95, 1.09) p=0.63 | 1 (1.09, 0.94) p=0.93 |
|  | Proportion of patients promptly mobilised (70% or less vs Between 70 and 80%) | 1.02 | (0.92, 1.13) | 0.726 | KPI | 1.01 (0.91, 1.11) p=0.86 | 0.99 (1.11, 0.91) p=0.9 |
|  | Proportion of patients promptly mobilised (More than 80% vs Between 70 and 80%) | 1.05 | (0.97, 1.15) | 0.207 | KPI | 1.07 (0.99, 1.16) p=0.08 | 1.06 (1.16, 0.99) p=0.08 |
| Governance | Social worker attends clinical governance meeting (Yes vs no/not stated) | 1.13 | (0.97, 1.30) | 0.107 | NHFD FA | 1.3 (1.13, 1.48) p<0.001 | 1.1 (1.48, 0.98) p=0.12 |

OR>1 indicates more likely to have worse mobility.

*Organisational factors are adjusted for case-mix (age, sex, ASA classification, hip fracture type, pre-fracture residence, and pre-fracture mobility) and mutually adjusted for all backward selected factors in table.

A1=stable, A2=unstable, ASA=American Society of Anesthesiologists, CI=confidence interval, ED=Emergency Department, FA=Facilities Audit, FTE=full time equivalent, NHFD=National Hip Fracture Database, NICE=National Institute for Clinical Excellence, op=operative.

Additional table 4: Baseline (case-mix) pre-fracture residence and mobility compared to these outcomes at 120 days and missingness, N=147,027

|  | | Residence at 120 days | | | |  | Mobility at 120 days | | | |
| --- | --- | --- | --- | --- | --- | --- | --- | --- | --- | --- |
|  | | **Recorded** | | **Missing** | |  | **Recorded** | | **Missing** | |
| **Pre-fracture characteristics** | | **N** | **%** | **N** | **%** |  | **N** | **%** | **N** | **%** |
| Total |  | 58,344 | 40 | 88,683 | 60 |  | 56,959 | 39 | 90,068 | 61 |
| Age (years) |  | Mean=82.1, SD=8.5 | | Mean=81.9, SD=8.7 | |  | Mean=82.1,  SD=8.5 | | Mean=81.9, SD=8.7 | |
| Age (years) | 60-69 | 5,541 | 9 | 9,366 | 11 |  | 5,376 | 9 | 9,531 | 11 |
|  | 70-79 | 14,483 | 25 | 22,105 | 25 |  | 14,159 | 25 | 22,429 | 25 |
|  | 80-89 | 26,820 | 46 | 39,622 | 45 |  | 26,209 | 46 | 40,233 | 45 |
|  | 90+ | 11,500 | 20 | 17,590 | 20 |  | 11,215 | 20 | 17,875 | 20 |
| Sex | Female | 42,627 | 73 | 64,170 | 72 |  | 41,637 | 73 | 65,160 | 72 |
| ASA grade* | I & II | 17,166 | 29 | 25,755 | 29 |  | 16,772 | 29 | 26,149 | 29 |
|  | III | 33,556 | 58 | 51,135 | 58 |  | 32,770 | 58 | 51,921 | 58 |
|  | IV & V | 7,622 | 13 | 11,793 | 13 |  | 7,417 | 13 | 11,998 | 13 |
| Hip fracture type | Intracapsular | 34,886 | 60 | 52,646 | 59 |  | 34,105 | 60 | 53,427 | 59 |
|  | Inter, subtrochanteric or other | 23,458 | 40 | 36,037 | 41 |  | 22,854 | 40 | 36,641 | 41 |
| Pre-fracture residence | Own home/sheltered housing | 49,392 | 85 | 76,043 | 86 |  | 48,207 | 85 | 77,228 | 86 |
|  | Not from own home | 8,952 | 15 | 12,640 | 14 |  | 8,752 | 15 | 12,840 | 14 |
| Pre-fracture mobility | Freely mobile without walking aids | 23,777 | 41 | 36,353 | 41 |  | 23,287 | 41 | 36,843 | 41 |
|  | Mobile outdoors with 1 or 2 aids or frame | 20,174 | 35 | 34,404 | 39 |  | 19,675 | 35 | 34,903 | 39 |
|  | Some indoor, or no functional, mobility | 14,393 | 25 | 17,926 | 20 |  | 13,997 | 25 | 18,322 | 20 |

ASA=American Society of Anesthesiologists, SD=standard deviation.

*I & II (healthy patient or patient with mild systemic disease), III (patient with a severe but not incapacitating systemic disease), IV & V (a patient with an incapacitating disease that is also life threatening or a moribund patient not expected to live for 24 h with or without surgery)

Additional table 5: Missing outcome data, relative to available outcome data, by organisational factors, for the outcomes of residence at 120 days and mortality at 120 days

|  |  | **Residence at 120 days (N=147,027 i.e. Alive at 120 days)** | | | | | **Mortality 120 days (N=165,350 i.e. survived superspell)** | | | | |
| --- | --- | --- | --- | --- | --- | --- | --- | --- | --- | --- | --- |
| **Organisational factor** | **Category** | **N with residence data** | **% with residence data** | **N without residence data** | **% without residence data** | **% with minus % without data** | **N Alive** | **% Alive** | **N Dead** | **% Dead** | **% alive minus % dead** |
| Consultant orthopaedic surgeon attends the daily trauma meeting | no/not stated | 2,591 | 4 | 7,156 | 8 | -4 | 9,747 | 7 | 1,292 | 7 |  |
|  | Yes | 55,753 | 96 | 81,527 | 92 | 4 | 137,280 | 93 | 17,031 | 93 |  |
| Dedicated hip fracture ward to which patients can be admitted direct from ED | no/not stated | 19,862 | 34 | 32,430 | 37 | -3 | 52,292 | 36 | 6,316 | 34 | 1 |
|  | Yes | 38,482 | 66 | 56,253 | 63 | 3 | 94,735 | 64 | 12,007 | 66 | -1 |
| Treatment plan routinely discussed with the patient and NOK on admission | no/not stated | 15,948 | 27 | 30,343 | 34 | -7 | 46,291 | 31 | 5,713 | 31 |  |
|  | Yes | 42,396 | 73 | 58,340 | 66 | 7 | 100,736 | 69 | 12,610 | 69 |  |
| Protocol in place for pre-op. energy supplement juice drinks | no/not stated | 33,919 | 58 | 50,980 | 57 | 1 | 84,899 | 58 | 10,717 | 58 | -1 |
|  | Yes | 24,425 | 42 | 37,703 | 43 | -1 | 62,128 | 42 | 7,606 | 42 | 1 |
| Proportion of patients given a nerve block pre-op. | 50% or less | 27,290 | 47 | 51,783 | 58 | -12 | 79,073 | 54 | 10,141 | 55 | -2 |
|  | More than 50% | 31,054 | 53 | 36,900 | 42 | 12 | 67,954 | 46 | 8,182 | 45 | 2 |
| Protocol in place for pre-op. care bundle | no/not stated | 3,855 | 7 | 12,943 | 15 | -8 | 16,798 | 11 | 2,066 | 11 |  |
|  | Yes | 54,489 | 93 | 75,740 | 85 | 8 | 130,229 | 89 | 16,257 | 89 |  |
| Proportion of eligible patients receiving a total hip replacement | 22% or less | 10,202 | 17 | 25,386 | 29 | -11 | 35,588 | 24 | 4,475 | 24 |  |
|  | More than 22% | 48,142 | 83 | 63,297 | 71 | 11 | 111,439 | 76 | 13,848 | 76 |  |
| Proportion of general anaesthetics accompanied with a nerve block | 50% or less | 16,897 | 29 | 22,368 | 25 | 4 | 39,265 | 27 | 4,937 | 27 |  |
|  | More than 50% | 41,447 | 71 | 66,315 | 75 | -4 | 107,762 | 73 | 13,386 | 73 |  |
| Anaesthetic rota protocol includes consecutive trauma theatre days on call | no/not stated | 33,617 | 58 | 45,197 | 51 | 7 | 78,814 | 54 | 9,794 | 53 |  |
|  | Yes | 24,727 | 42 | 43,486 | 49 | -7 | 68,213 | 46 | 8,529 | 47 |  |
| Proportion of trochanteric fractures receiving a sliding hip screw | 70% or less | 16,247 | 28 | 24,955 | 28 |  | 41,202 | 28 | 5,067 | 28 |  |
|  | More than 70% | 42,097 | 72 | 63,728 | 72 |  | 105,825 | 72 | 13,256 | 72 |  |
| Proportion of patients receiving an inpatient delirium assessment | less than 100% | 42,879 | 73 | 70,162 | 79 | -6 | 113,041 | 77 | 14,051 | 77 |  |
|  | 100% | 15,465 | 27 | 18,521 | 21 | 6 | 33,986 | 23 | 4,272 | 23 |  |
| Plans in place to reconfigure the local hip fracture service | no/not stated | 49,466 | 85 | 72,295 | 82 | 3 | 121,761 | 83 | 15,097 | 82 |  |
|  | Yes | 8,878 | 15 | 16,388 | 18 | -3 | 25,266 | 17 | 3,226 | 18 |  |
| Community rehab. team attends clinical governance meeting | no/not stated | 50,798 | 87 | 82,319 | 93 | -6 | 133,117 | 91 | 16,580 | 90 |  |
|  | Yes | 7,546 | 13 | 6,364 | 7 | 6 | 13,910 | 9 | 1,743 | 10 |  |

ED=Emergency Department, NOK=next of kin, op=operative, rehab.=rehabilitation.

Additional table 6: Missing outcome data, relative to available outcome data, by organisational factors, for the outcomes of mobility at 120 days and mortality at 120 days

| **Organisational factor** | **Category** | **Mobility at 120 days (N=147,027 i.e. Alive at 120 days)** | | | | | **Mortality 120 days (N=165,350 i.e. survived superspell)** | | | | |
| --- | --- | --- | --- | --- | --- | --- | --- | --- | --- | --- | --- |
|  |  | **N with mobility data** | **% with mobility data** | **N without mobility data** | **% without mobility data** | **% with minus % without mobility data** | **N Alive** | **% Alive** | **N Dead** | **% Dead** | **% Alive minus %Dead** |
| Pre-op. pain is routinely scored | no/not stated | 6,716 | 12 | 10,904 | 12 |  | 17,620 | 12 | 2,032 | 11 | 1 |
|  | Yes | 50,243 | 88 | 79,164 | 88 |  | 129,407 | 88 | 16,291 | 89 | -1 |
| Total FTE for ED consultants at the trust | 17 or less | 43,824 | 77 | 68,102 | 76 | 1 | 111,926 | 76 | 14,186 | 77 | -1 |
|  | More than 17 | 13,135 | 23 | 21,966 | 24 | -1 | 35,101 | 24 | 4,137 | 23 | 1 |
| Number of hours from admission to operation | 35 hours or less | 45,657 | 80 | 67,523 | 75 | 5 | 113,180 | 77 | 14,111 | 77 |  |
|  | More than 35 hours | 11,302 | 20 | 22,545 | 25 | -5 | 33,847 | 23 | 4,212 | 23 |  |
| Total FTE for non-consultant grade ED doctors at the trust | between 25 and 44 | 31,536 | 55 | 42,642 | 47 | 9 | 74,178 | 50 | 9,411 | 51 | -1 |
|  | 25 or less | 14,428 | 25 | 20,483 | 23 | 2 | 34,911 | 24 | 4,362 | 24 |  |
|  | More than 44 | 10,995 | 19 | 26,943 | 30 | -11 | 37,938 | 26 | 4,550 | 25 | 1 |
| Proportion of patients assessed by an orthogeriatrician within 72 hours of admission | less than 100% | 55,756 | 98 | 89,014 | 99 | -1 | 144,770 | 98 | 18,072 | 99 |  |
|  | 100% | 1,203 | 2 | 1,054 | 1 | 1 | 2,257 | 2 | 251 | 1 |  |
| Anaesthetic rota protocol includes consecutive trauma theatre days on call | no/not stated | 32,769 | 58 | 46,045 | 51 | 6 | 78,814 | 54 | 9,794 | 53 |  |
|  | Yes | 24,190 | 42 | 44,023 | 49 | -6 | 68,213 | 46 | 8,529 | 47 |  |
| Proportion of surgery which is NICE compliant surgery | 80% or less | 42,141 | 74 | 70,874 | 79 | -5 | 113,015 | 77 | 13,983 | 76 | 1 |
|  | More than 80% | 14,818 | 26 | 19,194 | 21 | 5 | 34,012 | 23 | 4,340 | 24 | -1 |
| Proportion of general anaesthetics accompanied with a nerve block | 50% or less | 16,366 | 29 | 22,899 | 25 | 3 | 39,265 | 27 | 4,937 | 27 |  |
|  | More than 50% | 40,593 | 71 | 67,169 | 75 | -3 | 107,762 | 73 | 13,386 | 73 |  |
| Proportion of trochanteric fractures receiving a sliding hip screw | 70% or less | 15,905 | 28 | 25,297 | 28 |  | 41,202 | 28 | 5,067 | 28 |  |
|  | More than 70% | 41,054 | 72 | 64,771 | 72 |  | 105,825 | 72 | 13,256 | 72 |  |
| Proportion of times surgery supervised by consultant surgeon and anaesthetist | 60% or less | 25,837 | 45 | 45,840 | 51 | -6 | 71,677 | 49 | 8,725 | 48 | 1 |
|  | More than 60% | 31,122 | 55 | 44,228 | 49 | 6 | 75,350 | 51 | 9,598 | 52 | -1 |
| Near-patient haemoglobin testing in routine use in recovery | no/unrecorded | 25,591 | 45 | 50,875 | 56 | -12 | 76,466 | 52 | 9,728 | 53 | -1 |
|  | Yes | 31,368 | 55 | 39,193 | 44 | 12 | 70,561 | 48 | 8,595 | 47 | 1 |
| Hours of orthogeriatric support time by specialist nurse | none or missing | 17,494 | 31 | 39,144 | 43 | -13 | 56,638 | 39 | 7,017 | 38 |  |
|  | 1 or more hours | 39,465 | 69 | 50,924 | 57 | 13 | 90,389 | 61 | 11,306 | 62 |  |
| Postop. pain is routinely scored on day 1 | no/not stated | 7,820 | 14 | 15,346 | 17 | -3 | 23,166 | 16 | 2,770 | 15 | 1 |
|  | Yes | 49,139 | 86 | 74,722 | 83 | 3 | 123,861 | 84 | 15,553 | 85 | -1 |
| Nursing lead attends clinical governance meeting | no/not stated | 6,507 | 11 | 17,453 | 19 | -8 | 23,960 | 16 | 3,171 | 17 | -1 |
|  | Yes | 50,452 | 89 | 72,615 | 81 | 8 | 123,067 | 84 | 15,152 | 83 | 1 |
| NHFD data are regularly disseminated to hip fracture ward staff | no/not stated | 6,622 | 12 | 17,355 | 19 | -8 | 23,977 | 16 | 3,022 | 16 |  |
|  | Yes | 50,337 | 88 | 72,713 | 81 | 8 | 123,050 | 84 | 15,301 | 84 |  |
| Clinical governance meetings occur monthly | no/not stated | 20,834 | 37 | 28,366 | 31 | 5 | 49,200 | 33 | 6,147 | 34 |  |
|  | Yes | 36,125 | 63 | 61,702 | 69 | -5 | 97,827 | 67 | 12,176 | 66 |  |
| Social worker attends clinical governance meeting | no/not stated | 54,539 | 96 | 88,767 | 99 | -3 | 143,306 | 97 | 17,861 | 97 |  |
|  | Yes | 2,420 | 4 | 1,301 | 1 | 3 | 3,721 | 3 | 462 | 3 |  |
| Consultant orthopaedic surgeon attends clinical governance meeting | no/not stated | 6,713 | 12 | 15,255 | 17 | -5 | 21,968 | 15 | 2,786 | 15 |  |
|  | Yes | 50,246 | 88 | 74,813 | 83 | 5 | 125,059 | 85 | 15,537 | 85 |  |
| Consultant orthogeriatrician attends clinical governance meeting | no/not stated | 9,657 | 17 | 27,395 | 30 | -13 | 37,052 | 25 | 4,810 | 26 | -1 |
|  | Yes | 47,302 | 83 | 62,673 | 70 | 13 | 109,975 | 75 | 13,513 | 74 | 1 |
| Consultant anaesthetist attends the daily trauma meeting | no/not stated | 26,420 | 46 | 51,263 | 57 | -11 | 77,683 | 53 | 9,758 | 53 |  |
|  | Yes | 30,539 | 54 | 38,805 | 43 | 11 | 69,344 | 47 | 8,565 | 47 |  |
| Hours of orthogeriatric consultant direct clinical care per week | 20 hours or less | 30,935 | 54 | 54,514 | 61 | -6 | 85,449 | 58 | 10,719 | 59 |  |
|  | More than 20 hours | 26,024 | 46 | 35,554 | 39 | 6 | 61,578 | 42 | 7,604 | 41 |  |

Rounding means that some percentages may not total 100.

ED=Emergency Department, FTE=full time equivalent, NHFD=National Hip Fracture Database, NICE=National Institute for Clinical Excellence, op=operative.

Additional figure 1: Flow diagram showing data linkage and derivation of study cohort

PEDW data received from NWIS

01/04/2013—31/03/2020

N = 19,396 patients

HES data received from NHS Digital

01/04/2013—31/03/2020

N = 219,885 patients

HES=Hospital Episode Statistics, NHS=National Health Service, ONS=Office for National Statistics, NHFD=National Hip Fracture Database, PEDW=Patient Episode Database for Wales, NWIS=NHS Wales Informatics Service.

**Died by 120 days N=18,323 patients**

**N=147,027**

Cohort with outcomes data:

Residence at 120 days N=58,344 patients

Mobility at 120 days N=56,959 patients

**N=****165,350**

Cohort with outcomes data:

Residence at discharge N=163,230 patients

HES and NHFD dataset linkage

N = 168,359 patients

PEDW and NHFD dataset linkage

N = 10,398 patients

**Died during superspell N=13,407 patients**

NHFD

Hip fracture patients admitted to an English, Welsh or Northern Ireland NHS hospital between 01/04/2016 and 31/03/2019

N = 188,084 ***events***

HES (with ONS)

Patients admitted to an English NHS hospital with a hip fracture between 01/04/2016 and 31/03/2020

N = 200,018 ***patients***

PEDW (with ONS)

Patients admitted to a Welsh NHS hospital with a hip fracture between 01/04/2016 and 31/03/2020

N = 17,542 ***patients***

**REDUCE patient dataset:**

HES-NHFD-PEDW

**N= 178,757 patients**

Additional figure 2: Venn diagram showing availability of outcome data for the 165,350 patients in the study cohort


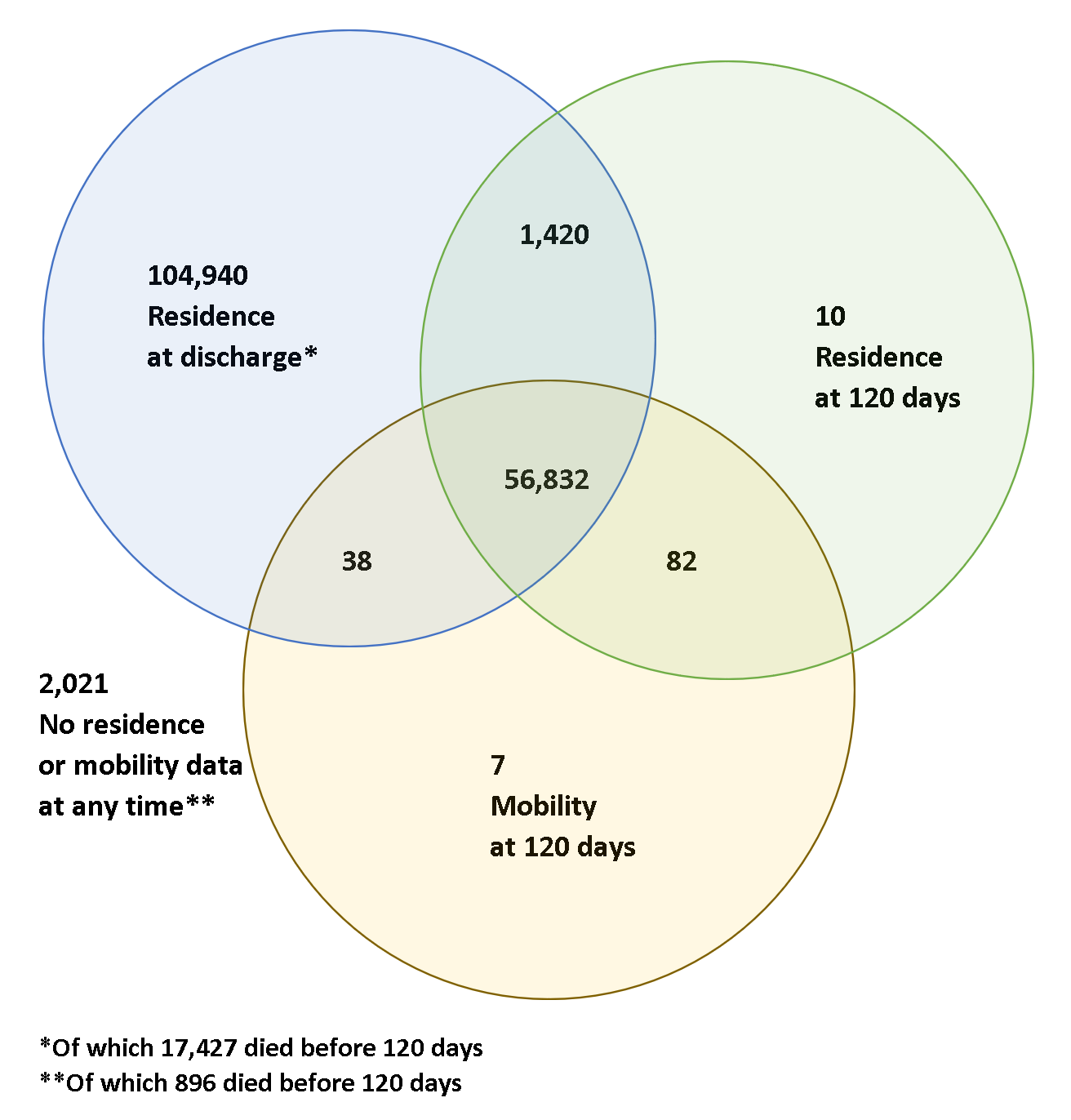


Additional figure 3: Percentage of patients returning to original residence at 120 days vs percentage of patients with missing 120 day residence data

N=58,340 patients with 120 day residence data at 139 hospitals (4 outlier hospitals excluded)

Proportion of patients returning to their original residence (of those with 120 day residence data) against the proportion missing 120 day residence data in each hospital (0.02, 95% CI: -0.02, 0.06).

Additional figure 4: Percentage of patients with same/improved mobility at 120 days vs percentage of patients with missing mobility data

N=56,950 patients with 120 day mobility data at 137 hospitals (3 outlier hospitals excluded)

Proportion of patients with good mobility outcomes (of those with mobility data at 120 days) against the proportion missing mobility data in each hospital (0.05, 95% CI: -0.03, 0.12).
